# Safety and Efficacy of Efpeglenatide in Patients With Type 2 Diabetes: A Meta-Analysis of Randomized Controlled Trials

**DOI:** 10.7759/cureus.45927

**Published:** 2023-09-25

**Authors:** Johao Escobar, Obinna Monday, Yashwanth Vemoori, Indresh Yadav, Abdalkareem Nael Jameel Maslamani, Salem Al Kutabi, Leena Saeed, Areeba Khan

**Affiliations:** 1 Medicine, American College of Physicians, Philadelphia, USA; 2 Medicine, Norfolk and Norwich University Teaching Hospital, Norwich, GBR; 3 Internal Medicine, Rajarajeswari Medical College and Hospital, Bengaluru, IND; 4 Internal Medicine, Samar Hospital and Research Center Pvt. Ltd., Janakpur, NPL; 5 Internal Medicine, Community Based Medical College Bangladesh, Mymensingh, BGD; 6 General Practice, Cairo University, Cairo, EGY; 7 Medicine, Cairo University, Cairo, EGY; 8 Internal Medicine, National Ribat University, Khartoum, SDN; 9 Critical Care Medicine, United Medical and Dental College, Karachi, PAK

**Keywords:** systematic review and meta-analysis, type 2 diabetes, efpeglenatide, safety, efficacy

## Abstract

The aim of this study was to assess the efficacy and safety of efpeglenatide in patients with type 2 diabetes (T2D). The study was reported according to the 2020 guidelines of the Preferred Reporting Items for Systematic Reviews and Meta-analyses (PRISMA) statement. Web of Science, PubMed, and Scopus databases were searched by two authors independently, with no restriction on language and year of publication, using the following key terms: (efpeglenatide) OR (glucagon-like peptide-1 receptor agonist) AND (type 2 diabetes) OR (diabetes) OR (T2DM) AND (HbA1c) OR (FSG) OR (fasting serum glucose) OR (weight) OR (bodyweight) OR (adverse events) OR (safety) OR (AE). Outcomes assessed in this meta-analysis included change in hemoglobin A1C (HbA1C) from baseline (%), change in weight from baseline (Kg), and change in fasting serum glucose (FSG) from baselines. For the safety analysis, we assessed total adverse events and gastrointestinal (GI) adverse events. A total of four studies fulfilled the inclusion and exclusion criteria and were included in this meta-analysis, encompassing six randomized controlled trials (RCTs). Compared with a control group, efpeglenatide lowered the HbA1c (mean difference (MD): -0.81, 95% confidence interval (CI): -1.01 to -0.60), body weight (MD: -1.15, 95% CI: -1.82 to -0.47), and FSG (MD: -0.98, 95% CI: -1.19 to -0.77). However, the risk of GI-related adverse events was significantly higher in the efpeglenatide group compared to the control group.

## Introduction and background

Glucagon-like peptide-1 receptor agonists (GLP-1RAs) are currently recommended as the initial injectable treatment for patients with type 2 diabetes (T2D) who did not achieve adequate control with oral glucose-lowering medications [1]. This recommendation is due to their effectiveness in controlling blood sugar levels, low risk of causing low blood sugar (hypoglycemia), and reduction in body weight [2]. Moreover, GLP-1RAs that have demonstrated cardiovascular benefits should be considered as a standalone therapy or in combination, regardless of a patient's initial hemoglobin A1C (HbA1C) levels or personalized HbA1c targets, for individuals with diabetes who are at a high risk of or already have atherosclerotic cardiovascular disease [3]. This recommendation is based on the evidence that certain GLP-1RAs, such as liraglutide taken once daily and others, including semaglutide, dulaglutide, and efpeglenatide taken once weekly, have shown cardiovascular and kidney benefits in this patient group [4-5]. In a study conducted by Gerstein et al., which enrolled individuals with T2D, a background of either prior cardiovascular disease or present kidney disease alongside at least one additional cardiovascular risk factor, the incidence of cardiovascular events was reduced in the group receiving weekly subcutaneous injections of efpeglenatide at either 4 or 6 mg doses compared to those who were administered a placebo [4].

Efpeglenatide is a long-acting GLP-1RA currently under development for the management of blood sugar levels in patients with T2D [6]. It is administered subcutaneously once weekly. Efpeglenatide is composed of a modified exendin molecule attached to a fragment of human immunoglobulin 4 using a special technology called long-acting peptide/protein (LAPS) [6]. This conjugation allows for flexible dosing frequencies, ranging from once weekly to once every two weeks or once monthly [7-8]. Efpeglenatide's superiority in signaling through the glucagon-like peptide-1 receptor (GLP-1R) and its reduced desensitization compared to other GLP-1RAs is a promising development in the field of diabetes treatment. This means that when efpeglenatide binds to the GLP-1R on the surface of pancreatic cells, it triggers more robust and sustained cellular responses [9]. This heightened signaling efficiency may lead to improved blood glucose control and better metabolic effects in patients with diabetes [9]. Moreover, the reduced desensitization suggests that efpeglenatide's effectiveness is less likely to diminish over time, potentially resulting in more consistent and durable therapeutic benefits compared to other GLP-1RAs. These findings raise hope for the development of more effective and enduring treatments for diabetes [9-10].

This meta-analysis has comprehensively evaluated efpeglenatide, a long-acting GLP-1 RA, and its potential impact on glycemic control and safety in T2D. Unique features, such as the novel conjugation technology and enhanced receptor signaling, make efpeglenatide a promising candidate deserving of critical appraisal within the existing landscape of diabetes therapeutics. By synthesizing existing research, this analysis seeks to provide valuable insights into efpeglenatide's role in optimizing the diabetes management for patients and healthcare practitioners. The aim of this meta-analysis is to assess the efficacy and safety of efpeglenatide in patients with T2D.

## Review

Methodology

The study was reported according to the 2020 guidelines of the Preferred Reporting Items for Systematic Reviews and Meta-analyses (PRISMA) statement.

Search Strategy

Web of Science, PubMed, and Scopus databases were searched by two authors independently, with no restriction on the language and year of publication, using the following key terms: (efpeglenatide) OR (glucagon-like peptide-1 receptor agonist) AND (type 2 diabetes) OR (diabetes) OR (T2DM) AND (HbA1c) OR (FSG) OR (fasting serum glucose) OR (weight) OR (bodyweight) OR (adverse events) OR (safety) OR (AE). The wild-card term "*" was utilized to enhance the sensitivity of the search strategy, which was confined to studies involving humans. The reference lists of the identified papers were manually inspected for any additional relevant articles. The literature search spanned from its inception to August 25, 2023.

Study Selection

Studies were included if they fulfill the following criteria: (1) were a randomized control trial with either a single center or multicenter design, (2) assessed the effect of efpeglenatide in patients with T2D irrespective of the dose and comparison group, and (3) assessed any of the outcomes evaluated in this meta-analysis. We excluded observational studies, reviews, editorials, and expert opinions. We also excluded studies that lacked a comparison group. All articles obtained from online database searching were thoroughly screened by two authors independently. Following the removal of duplicate entries, an initial screening process was conducted based on abstracts and titles. Subsequently, full-text versions of eligible records were acquired, and a thorough evaluation was carried out using predefined inclusion and exclusion criteria. Two authors independently reviewed the articles, and any differences in the assessment between them were resolved through collaborative discussions.

Data Extraction

Data abstracted from the included studies were author name, publication year, groups, dose, sample size, patients’ characteristics, and outcomes. Outcomes assessed in this meta-analysis included change in Hb1AC from baseline (%), change in weight from baseline (Kg), and change in fasting serum glucose (FSG) (mmol/L) from baselines. For the safety analysis, we assessed total adverse events and gastrointestinal (GI) adverse events.

Risk-of-Bias Assessment

Two authors (IW and SK) independently performed a thorough assessment of the potential for bias in the studies by using the Cochrane risk-of-bias tool. This assessment considered various factors, including whether the study had properly generated sequences, employed blinding, handled dropouts or missing data, concealed allocation, reported outcomes selectively, and accounted for other potential sources of bias. Each factor was categorized as presenting either a high, low, or unclear risk of bias. Any differences between the two authors' performed assessments were resolved through a consensus-based discussion.

Data Analysis

Data analysis was performed using RevMan Software version 5.4.1 (Cochrane Collaboration). Changes in continuous outcomes were calculated as mean difference (MD) with their 95% confidence interval (CI), while categorical outcomes (adverse events and GI-related events) were reported as a risk ratio (RR) with 95% CI. A meta-analysis was conducted using a random-effect model to deal with the variation. Heterogeneity was reported among the study as I^2^. We performed a subgroup analysis to compare three doses of efpeglenatide, i.e., 2 mg, 4 mg, and 6 mg.

Results

**Figure 1:**
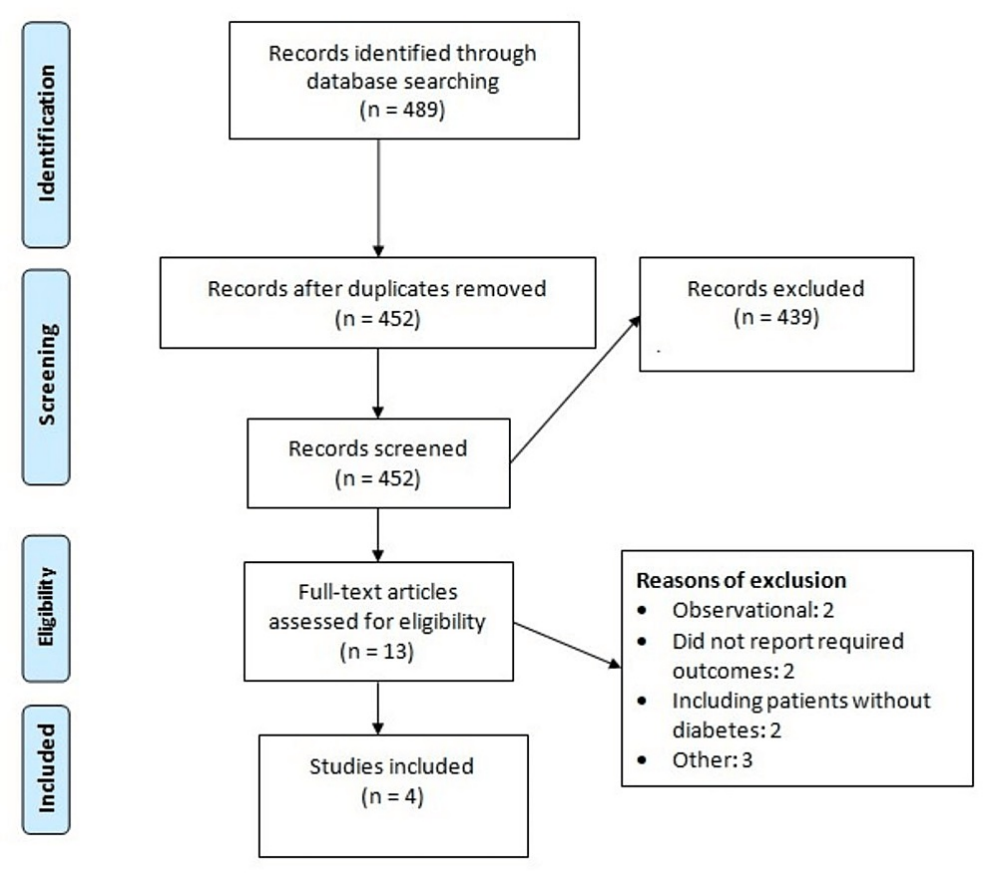
PRISMA flowchart showing the process of study selection

**Figure 2:**
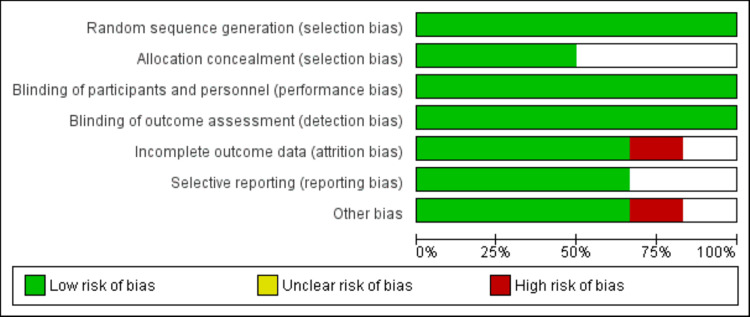
Risk-of-bias graph

Studies were selected by following the PRISMA guidelines. Figure 1 illustrates a flowchart outlining the process we followed to select the studies. An online database searching yielded to 489 studies. After removing duplicates, 452 studies were initially screened. In the end, four studies fulfilled the inclusion and exclusion criteria and were included in this meta-analysis. One study discussed three trials. All studies compared multiple doses of efpeglenatide, so we compared each dose of efpeglenatide separately. Table 1 shows the characteristics of the included studies. Figure 2 shows the quality assessment of the included studies.

**Table 1:** Characteristics of the included studies SD: standard deviation

Author	Year	Groups	Dose	Other drugs	Sample size	Follow-up	Mean age in years (SD)	Males (n)	Mean duration of diabetes in years (SD)
Amplitude D [11]	2023	Efpeglenatide	4 mg	Metformin	257	56 weeks	60.3 (9.6)	161	8.6 (6.36)
		Efpeglenatide	6 mg		254		60 (10.1)	145	8.8 (6.57)
		Control (dulaglutide)			250		59.4 (10.1)	150	8.6 (6.98)
Amplitude L [11]	2023	Efpeglenatide	2 mg	Metformin	92	30 weeks	59.1 (10.7)	50	15.4 (8.3)
		Efpeglenatide	4 mg		93		60.6 (11.5)	51	16.2 (7.3)
		Efpeglenatide	6 mg		93		61.6 (10.3)	53	16.3 (8.8)
		Control			92		58.9 (10.7)	43	15.2 (7.0)
Amplitude S [11]	2023	Efpeglenatide	2 mg	Metformin	78	30 weeks	60.1 (10.9)	44	10.6 (6.9)
		Efpeglenatide	4 mg		77		57.9 (10.5)	44	10.4 (7.4)
		Efpeglenatide	6 mg		78		58.8 (11.5)	39	11.5 (8.1)
		Control			79		58.9 (10.6)	45	9.5 (6.2)
Frias et al. [12]	2022	Efpeglenatide	2 mg	Oral antihyperglycemic drugs or insulin	100	30 weeks	58.6 (10.5)	55	5.3 (5.3)
		Efpeglenatide	4 mg		101		56.3 (11.5)	52	4.9 (5.0)
		Efpeglenatide	6 mg		103		59.6 (10.7)	61	5.2 (5.2)
		Control			102		59.5 (11.7)	51	5 (4.9)
Prato et al. [13]	2020	Efpeglenatide	8 mg	Metformin	52	17 weeks	56.7 (8.1)	19	9.1 (7.4)
		Efpeglenatide	12 mg		52		56 (9.5)	28	7.4 (6.2)
		Efpeglenatide	16 mg		52		56.4 (9.5)	25	7.2 (4.6)
		Control			49		54.7 (9.9)	23	7.2 (5.3)
Rosenstock (a) [14]	2019	Efpeglenatide	2 mg	Metformin	33	13 weeks	56 (10)	18	5.8 (5.2)
		Efpeglenatide	3 mg		36		54 (10)	23	5.9 (4.8)
		Efpeglenatide	4 mg		36		56 (10)	18	6.1 (6.4)
		Control			37		55 (9)	16	6.3 (5.1)

Meta-Analysis of Outcomes

**Figure 3:**
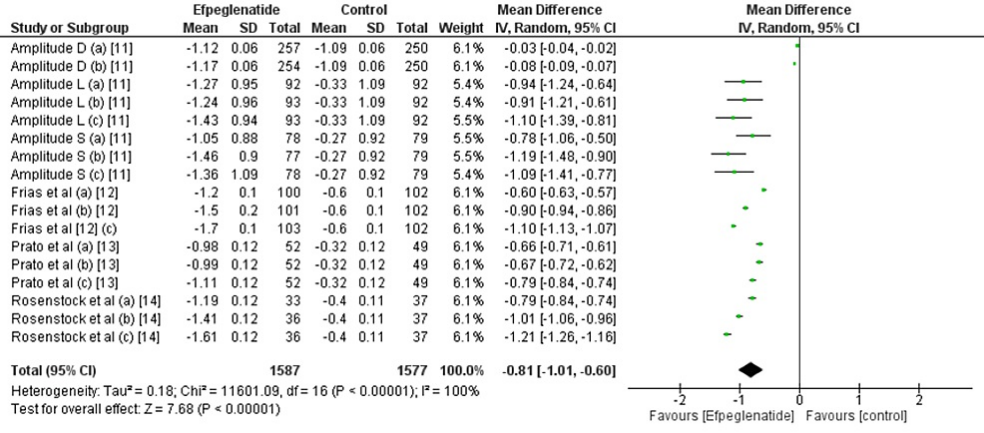
Comparison of HbA1C between groups Sources: References [11-14]

Change in HbA1C (%) from baseline: All the included studies reported the change in HbA1c from baseline. Compared with the control group, efpeglenatide lowered HbA1c significantly (MD: -0.81, 95% CI: -1.01 to -0.60), as shown in Figure 3. Based on the comparison of three doses of efpeglenatide, in all three doses, reduction in HbA1C was significantly greater in efpeglenatide compared to the control group, as shown in table 2. However, we found no significant difference among the effect of the three groups on the reduction in HbA1C (p-value<0.0001).

**Figure 4:**
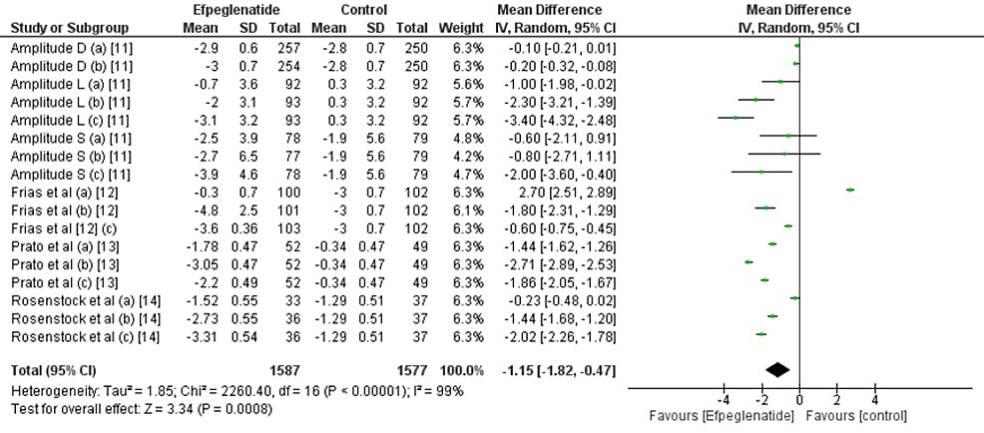
Comparison of body weight between groups Sources: References [11-14]

Change in body weight (kg): All the included studies reported a change in body weight from baseline. Compared with the control group, efpeglenatide lowered weight significantly (MD: -1.15, 95% CI: -1.82 to -0.47), as shown in Figure 4. In terms of weight, the reduction observed in the efpeglenatide 2 mg group did not show a significant difference when compared to the control group. However, in the efpeglenatide 4 mg and 6 mg groups, weight reduction is greater compared to the control group, as shown in Table 2. Overall, when considering the three efpeglenatide groups together, there is a significant difference in terms of weight reduction.

**Figure 5:**
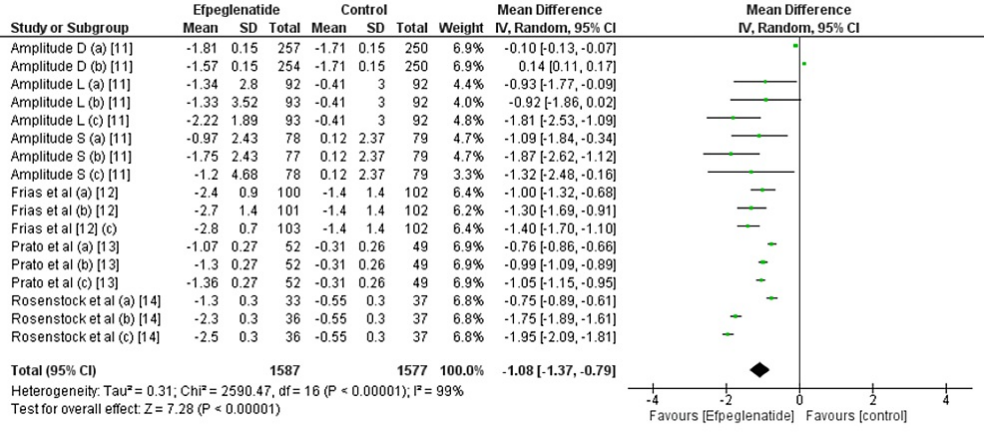
Comparison of FSG between groups Sources: References [11-14]

Change in FSG (mmol/L): All the included studies reported the change in FSG from baseline. As shown in Figure 5, reduction in FSG was significantly greater in patients randomized in the efpeglenatide group compared to the control group (MD: -0.98, 95% CI: -1.19 to -0.77). When comparing the three different doses of efpeglenatide, all of them demonstrated a significantly greater reduction in FSG levels compared to the control group, as indicated in Table 2. However, we observed no significant variance in the impact of the three groups on the reduction of FSG levels (p-value<0.0001).

**Table 2:** Subgroup analysis comparing the three doses of efpeglenatide MD: mean difference; FSG: fasting serum glucose; HbA1C: glycated hemoglobin

Outcomes	Subgroups	MD (95% CI)	P-value	P-value (sub-group differences)
HbA1C	2 mg	-0.75 (-0.90 to -0.59)	0.0001	0.99
	4 mg	-0.73 (-0.99 to -0.48)	0.0001	
	6 mg	-0.72 (-1.03 to -0.40)	0.0001	
Weight	2 mg	0.26 (-1.80 to 2.32)	0.8	0.04
	4 mg	-2.31 (-5.01 to -0.39)	0.001	
	6 mg	-2.73 (-5.65 to -0.20)	0.0001	
FSG	2 mg	-0.80 (-0.93 to -0.68)	0.0001	0.56
	4 mg	-1.01 (-1.36 to -0.66)	0.0001	
	6 mg	-0.83 (-1.29 to -0.37)	0.0004	

Safety Analysis

Table 3 presents the safety analysis of efpeglenatide in comparison to the control group. There were no significant differences observed between efpeglenatide and the control group in terms of total adverse events (relative risk (RR): 1.06, 95% CI: 0.98 to 1.15). The subgroup analysis yielded results consistent with the pooled analysis. Furthermore, GI-related adverse events were significantly more frequent in the efpeglenatide group compared to the control group (RR: 1.59, 95% CI: 1.18 to 2.14). Subgroup analyses confirmed the consistency of these results with the pooled analysis, and no significant differences were reported among the subgroups.

**Table 3:** Safety outcomes comparison between the groups RR: risk ratio; CI: confidence interval; GI: gastrointestinal

Outcomes	Group	RR (95% CI)	P-value	P-value (sub-group differences)
All adverse events	All groups	1.06 (0.98 to 1.15)	0.13	
	2 mg	1.03 (0.93 to 1.14)	0.58	0.45
	4 mg	1.03 (0.97 to 1.09)	0.53	
	6 mg	1.05 (0.95 to 1.17)	0.36	
GI-related adverse events	All groups	1.59 (1.18 to 2.14)	0.002	
	2 mg	1.37 (1.07 to 1.75)	0.01	0.32
	4 mg	1.44 (1.12 to 1.87)	0.005	
	6 mg	1.99 (1.60 to 2.48)	0.0001	

Discussion

As far as our knowledge is concerned, this is the first meta-analysis assessing the efficacy and safety of efpeglenatide in patients with T2D. The findings of this meta-analysis suggest that efpeglenatide is effective in reducing HbA1C, FSG, and weight. However, studies included in this review compared multiple doses of efpeglenatide with placebo. Therefore, we conducted a subgroup analysis to compare the effect of multiple doses (2, 4, and 6 mg) on the efficacy outcomes. Efpeglenatide, administered as a once-weekly injection, has demonstrated sustained and consistent activation of GLP-1Rs, leading to prolonged glucose-lowering effects in clinical studies. Unlike some other GLP-1RAs, which require more frequent dosing, efpeglenatide's extended dosing interval suggests the potential for enhanced treatment adherence and convenience [15]. However, these findings need to be validated in the future studies, and we need more studies that compared different GLP-1RAs to compare their efficacy and safety in T2D patients.

All included studies comparing efpeglenatide and placebo in T2D patients favor efpeglenatide in terms of reduction of HbA1C (%). In three trials, namely, Amplitude D, Amplitude L and Amplitude S [11], enrollment was closed early due to the decision of the sponsor related to the study funding and not due to any issues regarding the efficacy or safety of drugs. Although these trials were terminated early, their findings favor efpeglenatide with meaningful reductions in HbA1C, FPG, and body weight. As shown in Table 1, participants in the Amplitude L and Amplitude S trials have the longest duration of diabetes among all the included studies. It shows that the despite the differences in the disease characteristics of participants enrolled, the improvement in weight reduction and glycemic control variables was consistently seen across all the studies at all doses. The study conducted by Prato et al. [13], which incorporated monthly dosing of efpeglenatide, provided compelling evidence regarding its effectiveness in managing T2D. Specifically, it showcased remarkable outcomes in terms of its influence on HbA1c levels and body weight. In addition, the study underscored efpeglenatide's remarkable safety profile, affirming its viability as a therapeutic option for individuals with diabetes. However, it is noteworthy that the majority of studies on efpeglenatide have predominantly focused on weekly administration. While these studies have demonstrated its efficacy, the concept of monthly administration carries intrinsic advantages, particularly in the context of managing T2D, which is characterized by its chronic nature and the long-term commitment required for effective treatment [16-18]. Further studies are required to compare the monthly and weekly dosing of efpeglenatide in patients with T2D to guide clinical practice guidelines.

The safety profile of efpeglenatide was consistent with that of the GLP-1RA class [19]. Notably, GI events were the most commonly reported treatment-emergent adverse events (TEAEs). The findings from our meta-analysis also underscored that GI-related adverse events occurred more frequently in the efpeglenatide group than in the control group. This trend was consistent across various analyses, indicating a robust association. Furthermore, our investigation revealed that the incidence of GI-related events increased with the efpeglenatide dose level, aligning with the anticipated response for this drug class. All of the included studies reported that risks of GI-adverse events were higher in the efpeglenatide group [12-14]. However, the study comparing dulaglutide with efpeglenatide did not show any significant difference in GI-related adverse events. Similar effects have been reported with the long-acting GLP-1RA. Indirect comparisons with weekly, long-acting GLP-1RAs suggest that rates with efpeglenatide once monthly were comparable to those seen with semaglutide once weekly but higher versus dulaglutide once weekly [20-21]. However, it is important to note that the increase was significant. Importantly, these events were generally of mild to moderate severity and tended to resolve over time [10]. This information is significant in providing a comprehensive understanding of the safety profile of efpeglenatide, demonstrating that any potential GI-related concerns are manageable and transient. Overall, our safety analysis reaffirms that efpeglenatide maintains a favorable safety profile, with GI events as the primary TEAEs of note. These findings contribute to the body of knowledge surrounding GLP-1RAs and provide valuable insights for healthcare professionals and patients considering the use of efpeglenatide.

Biochemical and preclinical studies indicate that efpeglenatide possesses distinctive receptor characteristics that might account for its superior maximal GLP-1R activation and decreased loss of sensitivity compared to other GLP-1RAs when subjected to prolonged exposure in laboratory settings [9,18]. Efpeglenatide exhibits faster dissociation kinetics from the GLP-1R in contrast to its counterparts among GLP-1RAs. This property enables efpeglenatide to function as a full agonist for triggering insulin secretion in response to glucose while concurrently reducing the extent of agonist-induced receptor internalization. Consequently, this results in a greater availability of receptors on the cell surface with extended exposure in vitro [9]. Whether these unique receptor characteristics will translate into more effective weight loss outcomes for patients compared to other GLP-1RAs remains a subject for further investigation.

The current meta-analysis has certain limitations. First, only one study used a monthly dose of efpeglenatide; therefore, we were not able to perform an analysis comparing the weekly and monthly administrations of efpeglenatide in terms of adherence, safety, and efficacy. However, we conducted a subgroup analysis based on the dose of efpeglenatide (2 mg, 4 mg, and 6 mg). Second, there are not many studies comparing efpeglenatide with other GLP-1 inhibitors. In addition, unpublished works, such as preprints, were not included in this meta-analysis. Lastly, one of the trials were not included in this meta-analysis as we were unable to extract required information from the paper. However, we contacted the author of that but could not got any response. The findings of that study were similar to our meta-analysis showing efficacy of efpeglenatide in patients with T2D [19]. In the future, more trials are needed to compare efpeglenatide with other GLP-1 inhibitors to determine the optimum therapy for patients with T2D.

## Conclusions

This meta-analysis, comprising six RCTs, found that efpeglenatide is significantly effective in reducing HbA1C levels, FSG, and body weight when compared to a placebo. It is worth noting, however, that the risk of GI-related adverse events was more common among patients treated with efpeglenatide. Furthermore, the findings from this meta-analysis revealed that the reduction in HbA1C and FSG levels remained significant across all doses of efpeglenatide studied. These results suggest a consistent and dose-dependent effect of efpeglenatide in improving glycemic control. To refine clinical practice guidelines and gain a more comprehensive understanding of efpeglenatide's role in the management of T2D, further research is warranted. This could include head-to-head comparisons with other GLP-1 inhibitors and exploration of the potential benefits of monthly dosing schedules. Such investigations will contribute to a more robust assessment of efpeglenatide's place in the management of T2D.
